# Natural and Synthetic Compounds for Management, Prevention and Treatment of Obesity

**DOI:** 10.3390/ijms23052890

**Published:** 2022-03-07

**Authors:** Antonella D’Anneo, Marianna Lauricella

**Affiliations:** 1 Laboratory of Biochemistry, Department of Biological, Chemical and Pharmaceutical Sciences and Technologies (STEBICEF), University of Palermo, 90127 Palermo, Italy; 2 Department of Biomedicine, Neurosciences and Advanced Diagnostics (BIND), Institute of Biochemistry, University of Palermo, 90127 Palermo, Italy

For a long time, adipose tissue has been considered an inert tissue involved in fat accumulation. Indeed, this tissue acts as an energy reserve, synthesizing and storing triacylglycerols from an excess of lipids and carbohydrates in feeding conditions (or degrading them in fasting ones). However, over the last few decades, emerging evidences has highlighted that adipose tissue serves as endocrine organ which is able to synthesize and secrete a significant amount of hormones, cytokines, and enzymes [1]. The substances produced and released by adipose tissue are known as adipokines and are able to act locally through autocrine/paracrine mechanisms or to influence the response of many organs and tissues through endocrine mechanisms, including the hypothalamus, pancreas, liver, skeletal muscle, and immune system [2]. Adipokines are involved in the control of a range of processes, including food intake, insulin sensitivity, and inflammatory pathways [2].

In addition to adipocytes, adipose tissue is composed by other cell types including immune cells, endothelial cells, and adipose precursor cells (among others), which contribute to the production of adipokines and cytokines as a result of cellular crosstalk. Notably, immune cells which are associated or recruited in adipose tissue can release both pro- or anti-inflammatory cytokines whose production changes in relationship to the expansion of adipose tissue [3]. 

It is well known that adipose tissue is capable of expanding to accommodate increasing amounts of lipids through hypertrophy of existing adipocytes and by the production of new adipocytes from precursors (hyperplasia). Obese patients are characterized by an expansion of adipose tissue resulting from a combination of excessive food intake, inadequate lifestyle, reduced physical activity, and genetic predisposition [4,5]. Both increased size (hypertrophy) and increased number (hyperplasia) of adipocytes contribute to adipose tissue expansion in obesity. Hypertrophy and hyperplasia of adipose tissue leads to a low-grade systemic inflammation and macrophage infiltration inside the adipose tissue, which is correlated with an unbalanced release of adipokines with pro-inflammatory roles [6]. This contributes to the development of chronic diseases including insulin resistance, type II diabetes, non-alcoholic pancreatic steatosis, atherosclerosis, hypertension and, among others, some forms of cancer as well [7,8]. Therefore, it is evident that obesity represents today a worldwide health problem (also considering its increased prevalence in the last years). The rising global trend of obesity, particularly in high-income countries, partially results from social and economic changes, called “obesogenic” changes, including economic growth, growing availability of inexpensive and often nutrient-poor food, and urbanization [5]. A low caloric diet associated with an increased physical activity represents the first strategy to reduce the expansion of adipose tissue in obese patients. Drugs such as orlistat, lorcaserin, or naltrexone are employed in the treatment of severe obesity, but their use has been associated with possible side effects [9]. Thus, the knowledge of the underlying pathophysiological mechanisms of obesity and the identification of new compounds with anti-obesity effects represents a strategy for reducing obesity and its related comorbidities. Given these premises, when we were invited as guest editors of this Special Issue, we proposed to explore the potential role of natural and synthetic compounds to reduce or prevent obesity and related diseases, focusing in particular on the molecular mechanisms of their action.

Nowadays, emerging evidence suggests that the plant kingdom is a rich source of phytochemicals endowed with biological activities. The fruits, leaves, and roots of several plants contain bioactive compounds capable of exerting anti-oxidant, anti-inflammatory, and anti-cancer effects [10,11,12,13,14]. Recently, most of these compounds have also been shown to exert anti-obesity effects [15,16,17].

Essential oils (EOs) are volatile compounds present in the flower petals, exocarp, tree bark, and roots of aromatic and medicinal plants. EOs are a heterogeneous group of compounds whose major components are monoterpenes and sesquiterpenes. The review of De Blasio et al. [18] highlighted the beneficial effects of OEs and their specific components on counteracting and/or preventing obesity in both in vitro and in vivo models. In particular the authors describe how the anti-obesity effects of different EOs can be correlated with their ability to regulate lipogenesis and lipolysis, to modulate adipokines release, and to stimulate browning. They also showed how several EOs-derived compounds are capable of counteracting obesity-related diseases, including type-2 diabetes, hypertension, and cardiovascular diseases.

Another interesting plant-derived compound is curcumin, a polyphenolic compound isolated from *Curcuma longa* L. rhizome. The review of Kasprak-Drozd et al. [19] focused on the anti-obesity effects of curcumin. The analysis of the mode of action of this natural compound in both in vitro and in vivo models showed that curcumin is capable of suppressing pre-adipocyte differentiation, reducing lipogenesis, increasing energy expenditure, and counteracting inflammatory pathways. The elucidation of anti-adipogenic mechanism of action of curcumin highlighted that curcumin exerts a pleiotropic effect that includes the stimulation of Wnt signaling cascade, the down-regulation of lipogenic transcription factors as SREBP and PPARγ the reduction of inflammatory mediators such as TNF-α and IL-6, as well as the upregulation of activators of catabolic pathway such as AMPK and SIRT1. 

Another contribution provided by Chung and Hyun [20] focused on the ability of pinostilbene to counteract adipogenesis of 3T3-L1 adipocytes. Pinostilbene is a methylated derivative of resveratrol, a polyphenolic compound found in several peanuts or red grapes that shows clear anti-inflammatory, antioxidant, and anti-cancer effects. Unfortunately, the unstable structure of resveratrol reduces its bioavailability and bioactivity. Interestingly, the methylated derivative, pinostilbene, has shown similar biological effects to that of resveratrol but with higher stability. The study of Chung and Hyun demonstrated that pinostilbene reduced lipid accumulation in adipocytes and counteracted adipogenesis and lipogenesis. These effects seem to be mediated by the ability of the compound to inhibit MAPK- and AKT-dependent insulin signaling as well as to activate AMPK-dependent pathway.

Browning is a well-known process of trans-differentiation of white adipose cells into beige adipose cells, an intermediate cell type between white and brown adipocytes [21]. Whereas white adipose cells serve as an energy fat store, brown and beige cells, due to the presence of the uncoupling protein UCP1, dissipate energy as heat by uncoupling the mitochondrial respiratory chain. Thus, browning activation could represent a strategy to reduce obesity and related disorders. To this end, the paper of Cruciani et al. [22] focused on the ability of metformin to reduce inflammation during adipose-derived stem cells differentiation and to promote browning. Metformin is a hypoglycemic compound widely used to treat obesity-related diabetes. The authors demonstrated that metformin in combination with the natural compound vitamin D reduced the secretion of pro-inflammatory cytokines IL-6 and TNF-α during adipose-derived stem cells differentiation. Interestingly, the combined treatment also promoted the “browning” in adipose-derived stem cells by increasing the UCP-1 uncoupling protein.

Obesity and diabetes are important risk factors for the development of cardiovascular diseases as well as myocardial infarctions [23]. After ischemia or injury, the cardiac muscle undergoes to an aberrant fibrotic remodeling induced by activation of inflammatory pathways, which leads to cardiac decline. Intriguingly, Loi et al. [24] highlighted the metformin ability to attenuate post-infarction myocardic fibrosis and inflammation in mice. In particular, the authors demonstrated that metformin reduced macrophage accumulation and collagen production in mice, thus counteracting fibrosis remodeling after myocardic injury.

The epidemiological association between obesity and cardiometabolic disorders has been well documented (although its underlying pathophysiological mechanisms have been scarcely understood). In this regard, Ugwoke et al. [25] analyzed the intimate relationship existing between skeletal muscle microvascular dysfunction and insulin resistance in obesity. Different etiologic biochemical mechanisms including endothelial impairment, remodeling of extracellular matrix, induction of oxidative stress, and the immunoinflammatory phenotype (as well as the therapeutically relevant approaches to improve the clinical perspectives of obese patients) were discussed by the authors.

On the other hand, considering the complexity of mechanistic and functional aspects of adipose tissues, as well as the different epigenetics changes and signaling pathways that can modulate specific obesity-associated genes, researchers have also paid their attention to the search of natural or synthetic compounds or biologic molecules acting at this level [26]. Indeed, the identification of targeted drugs regulating signaling and epigenetic status in adipogenesis could shed light on the treatment of obesity or related metabolic diseases through modulating adipogenesis [26]. Potential candidates in this direction seem to be epigenetic modulators affecting histone acetylation such as C646, a specific CBP/p300 inhibitor that significantly decreased the adiposity in larval zebrafish [27], or histone deacetylase inhibitors that reduced lipid accumulation in cultured adipocytes [28] and caused loss of body weight [29]. Further studies in this direction have been conducted using small molecules such as resveratrol as sirtuin activators. In particular, promoting SIRT1 activity, resveratrol induced PGC1α and ameliorated insulin sensitivity in resveratrol-treated mice [30].

In this scenario, considering the escalating spread of obesity, metabolic disorders, and their complications, which make it a serious global health problem, the search for new early biomarkers and appropriate diagnostic methods to assess possible risks have become an urgent need. In line with these new research landscapes, Zyśk et al. [31] highlighted the importance of specific biological fluids as matrices which can be used to research new putative and early biomarkers. On this subject, the authors focused on saliva as a biological matrix, or an alternative to serum samples, for use in diagnosis of obesity or other metabolic disorders from the analysis of adipokine and cytokine content.
